# ^64^Cu-DOTATATE-PET/CT in Neuroborreliosis Shows Increased Tracer Uptake in Dorsal Root and Paravertebral Ganglia

**DOI:** 10.3390/diagnostics16040561

**Published:** 2026-02-13

**Authors:** Mathilde Ørbæk, Marie Øbro Fosbøl, Anna Maria Florescu, Christian Midtgaard Stenør, Micha Phill Grønholm Jepsen, Jonathan Frederik Carlsen, Christian Thomas Brandt, Pelle Trier Petersen, Helene Mens, Åse Bengaard Andersen, Flemming Littrup Andersen, Ian Law, Annika Loft, Andreas Kjaer, Anne-Mette Lebech

**Affiliations:** 1 Department of Infectious Diseases, Copenhagen University Hospital—Rigshospitalet, 2100 Copenhagen, Denmark; 2 Department of Clinical Physiology and Nuclear Medicine—Rigshospitalet, 2100 Copenhagen, Denmarkian.law@regionh.dk (I.L.);; 3 Department of Neurology, Herlev-Gentofte Hospital, 2730 Herlev, Denmark; 4 Department of Clinical Medicine, Faculty of Health and Medical Sciences, University of Copenhagen, 2200 Copenhagen, Denmark; 5 Department of Pulmonary and Infectious Diseases, North Zealand Hospital, 3400 Hillerød, Denmark; 6 Department of Radiology, Copenhagen University Hospital—Rigshospitalet, 2100 Copenhagen, Denmark; 7 Department of Medicine, Zealand University Hospital, 4000 Roskilde, Denmark; 8 Cluster for Molecular Imaging, Department of Biomedical Sciences, University of Copenhagen, 2200 Copenhagen, Denmark

**Keywords:** *Borrelia burgdorferi*, PET, ^64^Cu-DOTATATE, neuroborreliosis, dorsal root ganglia, MRI

## Abstract

**Background:** Macrophages play a key role in clearing *Borrelia burgdorferi* infection and express somatostatin receptor subtype 2 (SSTR2), a potential imaging target. This study investigates immune activation in neuroborreliosis (NB) and assesses the diagnostic value of ^64^Cu-DOTATATE positron emission tomography/computed tomography (PET/CT) alongside magnetic resonance imaging (MRI). **Methods:** Prospective cohort study (2024–2025) enrolling patients with suspected NB from four Danish hospitals. NB was defined by the following ≥2 criteria: neurological symptoms, cerebrospinal fluid (CSF) pleocytosis, and intrathecal *B. burgdorferi*-specific antibodies; patients not meeting these criteria served as controls. **Results:** The study included 20 participants: 15 NB patients (75%) and 5 controls (25%). PET/CT was performed after a median of 9.5 days of antibiotic treatment. Symmetric ^64^Cu-DOTATATE uptake in dorsal root and paravertebral ganglia was observed in 10 of 15 patients, with cervical involvement in 8 and lumbosacral in 9. All of them had symptoms that corresponded to the anatomical distribution of the uptake. No controls had lumbosacral involvement (*p* = 0.04). One control with erythema migrans and systemic symptoms showed cervical ganglia uptake. MRI showed cranial or spinal nerve enhancement in five patients. Only one patient had focal PET uptake matching MRI findings and clinical facial palsy. **Conclusions:** Symmetric ^64^Cu-DOTATATE ganglionic uptake in NB patients may reflect immune activation or altered ganglionic physiology. One patient had focal ^64^Cu-DOTATATE uptake corresponding with palsy and MRI and ^64^Cu-DOTATATE PET/CT did not contribute additional diagnostic value beyond standard clinical evaluation.

## 1. Introduction

The diagnosis and monitoring of neuroborreliosis (NB) remains challenging due to its reliance on clinical symptoms combined with cerebrospinal fluid (CSF) analysis, specifically CSF pleocytosis and intrathecal *Borrelia burgdorferi* (*B. burgdorferi*)-specific antibody production [1,2] CSF pleocytosis resolves within weeks after antibiotic treatment, but intrathecal *B. burgdorferi* antibodies may persist for years [3,4]. Common symptoms in adult NB patients include painful meningoradiculitis, lymphocytic meningitis, and cranial nerve palsy, and typically develop within two months of an infected tick bite [3,5,6]. Despite a recognizable clinical presentation, a diagnostic delay of two to three weeks has remained unchanged over decades [5,7]. Antibiotic treatment eradicates the pathogen and accelerates recovery, hereby preventing late-stage infection [2,8]. Despite generally favorable outcomes with standard treatment, approximately 30–40% of patients experience persistent symptoms six months after treatment. Studies suggest that a delayed diagnosis and high symptom severity at treatment onset increase this risk [5,6,9].

The ^64^Cu-labeled tracer, [1,4,7,10-tetraazacyclododecane-N,N′,N″,N‴-tetraacetic acid]-d-Phe^1^,Tyr^3^-octreotate (^64^Cu-DOTATATE), is currently used for diagnosis, monitoring, and treatment of patients with neuroendocrine tumors (NETs) that express somatostatin receptor subtype 2 (SSTR2) [10]. Given that macrophages also express SSTR2, the diagnostic potential of ^64^Cu-DOTATATE is being explored in inflammatory and infectious conditions. The uptake of SSTR2-targeting tracers has been demonstrated in infected heart valves in patients with infective endocarditis, in atherosclerotic plaques, and in patients with sarcoidosis [11,12,13]. Macrophages are crucial in the engulfing and degrading of *B. burgdorferi* [14,15], and in a preclinical murine model of Lyme arthritis, ^64^Cu-DOTATATE showed uptake in infected joints [16]. Magnetic resonance imaging (MRI) is primarily used to exclude differential diagnoses but may suggest NB through findings of leptomeningeal enhancement or enhancement of cranial nerves and nerve roots [17,18]. We hypothesized that ^64^Cu-DOTATATE PET/CT and MRI would improve the diagnostic accuracy of NB. Additionally, it may offer insight into *B. burgdorferi* pathogenesis by visualizing spirochetal dissemination, central nervous system (CNS) infection, and local immune responses.

## 2. Materials and Methods

### 2.1. Study Design and Population

The study was designed as a prospective observational cohort study. Adult participants (aged 18 years or older) clinically suspected of NB were recruited in 2024 from the following four Danish institutions: Department of Infectious Diseases, Copenhagen University Hospital—Rigshospitalet; Department of Infectious Diseases, Copenhagen University Hospital—North Zealand; Department of Neurology, Copenhagen University Hospital—Herlev and Gentofte; and Department of Infectious Diseases, Zealand University Hospital, Roskilde. Participants were excluded if they were pregnant or breastfeeding, had already completed antibiotic treatment (defined as 14 days after diagnosis), or exceeded the PET/CT and MRI scanner bed weight limit (140 kg). Additionally, participants with a history of allergic reactions to compounds chemically or biologically similar to ^64^Cu-DOTATATE, or any contraindications to MRI as determined by a standard checklist, were excluded from the study. Diagnosis was based on the European Federation of Neurological Societies (EFNSs) guidelines [1]. Patients with definite NB were required to meet all of the following three criteria: NB-associated neurological symptoms, CSF pleocytosis, and intrathecal production of *B. burgdorferi*-specific antibodies. Those meeting two criteria were classified as possible NB. Participants with clinical suspicion of NB, who did not meet the EFNS criteria for either definite or possible NB, were classified as controls.

### 2.2. Ethics

All participants provided both oral and written informed consent in accordance with the requirements of the National Ethics Committee. The study was approved by Forskningsjura Region Hovedstaden (P-2020-686) and the local ethics committee (H-20051925). The radioligand ^64^Cu-DOTATATE is used on a compassionate use approval. Additionally, the study was registered at ClinicalTrials.gov (NCT06392815).

### 2.3. Clinical Data

Study data were collected and managed using Research Electronic Data Capture (REDCap), a secure, web-based platform designed to support data collection, hosted at Region Hovedstaden. The following information was extracted from patient records: age, gender, performance status, weight, height, comorbidities, clinical characteristics, diagnostic work-up including CSF analysis, biochemistry, previous imaging, and current medication, including antibiotic therapy. At a clinical visit at the time of diagnosis, symptoms and quality of life were assessed using standardized questionnaires, including the Short Form (36) Health Survey (SF-36) and the Multidimensional Fatigue Inventory-20 (MFI-20). The SF-36 assesses health-related quality of life across the following eight domains: physical functioning, pain, limitations due to physical health problems, limitations due to emotional problems, emotional well-being, social functioning, energy and fatigue, and general health perceptions. Each domain is scored from 0 to 100, with higher scores indicating better health status. The MFI-20 measures fatigue across the following five dimensions: general fatigue, physical fatigue, mental fatigue, reduced motivation, and reduced activity. Each domain is scored from 4 to 20, with higher scores indicating greater fatigue. Cognitive function was screened using the Montreal Cognitive Assessment (MoCA), which has a maximum score of 30. NB patients had a follow-up clinical visit six months after diagnosis.

### 2.4. ^64^Cu-DOTATATE PET/CT

A dose of up to 200 MBq of ^64^Cu-DOTATATE, consistent with routine clinical practice for NETs, was administered in accordance with current department guidelines. A static whole-body 10 min PET/CT scan (Long axial field of view (LAFOV) Siemens Vision Quadra, Siemens, Erlagen, Germany) was performed 120 min post-injection. The 120 min uptake time is consistent with our institutional imaging protocol and is supported by prior evidence showing that lesion detection with ^64^Cu-DOTATATE remains stable across imaging performed between 1 and 3 h post-injection [19]. The PET/CT was scheduled within 14 days of treatment initiation and conducted at the Department of Clinical Physiology and Nuclear Medicine, Rigshospitalet. Whole-body images were reconstructed from vertex to mid-femur (1 bed position, 106 cm) using 3D-OP-OSEM with 4 iterations, 5 subsets, point spread function (PSF) modeling, and a 2 mm Gaussian post filter. The matrix size was 440 × 440 voxels at 1.65 mm × 1.65 mm and a 2 mm slice spacing. A dedicated brain reconstruction was performed with a 0.825 mm in-plane voxel size and no post-filtering. Both reconstructions were performed with a maximum ring difference of 322 (UHS mode).

### 2.5. MRI of the Brain and Spinal Cord

In a separate setting, the patients underwent MRI (General Electric, Signa 1.5T, GE HealthCare Technologies Inc., Chicago, IL, USA) at the Department of Radiology, Rigshospitalet. During the scan, an intravenous gadolinium-based contrast agent was administered. In participants with reduced kidney function, contrast administration was omitted, in accordance with local guidelines. To prevent interference from blood residues in the CSF, MRI was scheduled at least four days after lumbar puncture.

### 2.6. Interpretation of Scans

All ^64^Cu-DOTATATE PET/CT and MRI scans were evaluated individually and after rigid PET and MRI image alignment and fusion (MI Oncology, Syngo.via, Siemens, Erlagen, Germany). Evaluations were performed by a team consisting of an experienced specialist in clinical physiology/nuclear medicine and an experienced specialist in radiology. Nervous system foci with ^64^Cu-DOTATATE uptake were described and quantified using maximum and mean standardized uptake values (SUV_max_ and SUV_mean_), with SUV_max_ as the primary readout.

### 2.7. Endpoint

The primary endpoint was the diagnostic performance of ^64^Cu-DOTATATE PET/CT and MRI in detecting NB, measured by the difference in tracer uptake between NB patients and controls. The secondary endpoint was to explore imaging findings relevant to the pathogenesis of *B. burgdorferi*.

### 2.8. Statistical Analysis

The sample size estimation was based on preclinical data [16], with an expected SUV_max_ difference of 0.45 (standard deviation 0.5) between definite NB patients and controls. To achieve an 80% power with a 5% type I error, 39 participants were required. An interim analysis was performed after 15 NB patients completed both ^64^Cu-DOTATATE PET/CT and MRI. The study was terminated as interim results indicated that the primary objective—demonstrating diagnostic discrimination between NB and controls—was unlikely to be met. All data analyses were performed using SAS Statistical Software Enterprise Guide version 8.4 (SAS Institute Inc., Cary, NC, USA). A two-sided *p*-value below 0.05 was considered statistically significant. Categorical variables were reported as counts and percentages, and comparisons were conducted using Fisher’s Exact test. Continuous variables were reported as median values with interquartile ranges (IQRs). The Wilcoxon rank-sum test (Mann–Whitney U) was used for unpaired comparisons between NB patients and controls. Paired *t*-tests were used to assess changes over time.

## 3. Results

### 3.1. Baseline Characteristics

Despite a target sample size of 39 participants, the study was terminated early after enrolling 15 NB patients (10 definite, 5 possible) and 5 controls due to interim analysis results. Among controls, one had polymyalgia rheumatica, one had erythema migrans, and three had no specific diagnosis after thorough evaluation. Baseline characteristics are presented in Table 1. The median age was 56 years (IQR 48–66), and 35% were females. At inclusion, the majority (65%) reported symptoms lasting 15–44 days. The median duration of antibiotic treatment at the time of PET/CT were 9.5 (IQR 7–11) days. Common symptoms included radiating pain (50%), sensory disturbances (50%), fatigue (85%), muscle pain (67%), and dizziness (55%). Among NB patients, 33% reported facial or eye paralysis, 20% limb paresis, 27% changes in hearing or taste, and 33% experienced both radicular pain and paresis.

### 3.2. ^64^Cu-DOTATATE PET

**Figure 1:**
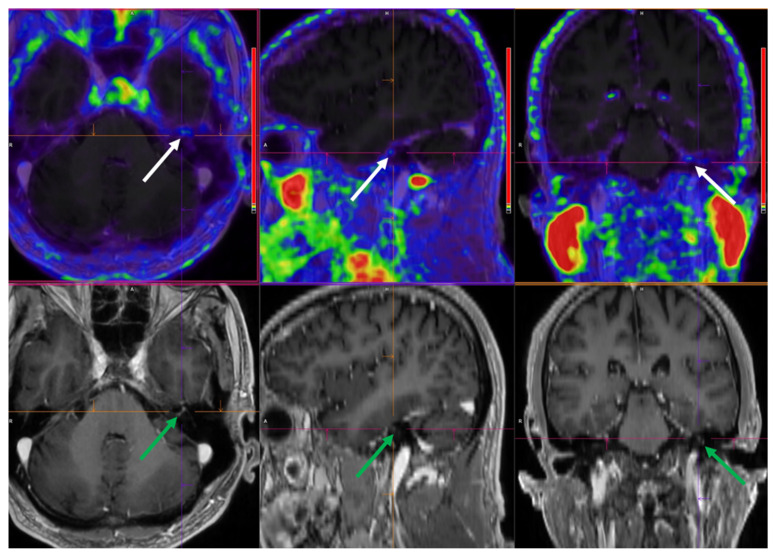
^64^Cu-DOTATATE PET fused with T1 post-contrast MRI in patient with neuroborreliosis. (Transaxial, sagittal and coronal view, respectively). Abbreviations: R = Right, H = Head, A = Anterior. Focal ^64^Cu-DOTATATE uptake in the left cranial facial nerve corresponds to MRI enhancement and left facial palsy symptoms. PET/CT images (**upper** rows) show discrete focal uptake of ^64^Cu-DOTATATE (SUV_max_ = 0.9) in the left cranial facial nerve (white arrows), corresponding to the contrast-enhanced segment observed on MRI (**lower** rows, green arrows).

Whole-body ^64^Cu-DOTATATE PET/CT was successfully performed in all participants. In one NB patient, faint focal ^64^Cu-DOTATATE uptake corresponded with cranial nerve enhancement on MRI and clinical facial palsy (Figure 1). Two NB patients showed intense focal meningeal ^64^Cu-DOTATATE uptake without corresponding MRI lesions—one near the petrous part of the temporal bone and the other close to the anterior sphenoid process. Despite not being visible on MRI, these lesions resembled incidental meningiomas and were considered unrelated to NB. No other ^64^Cu-DOTATATE-positive brain lesions suggestive of NB were detected.

**Figure 2:**
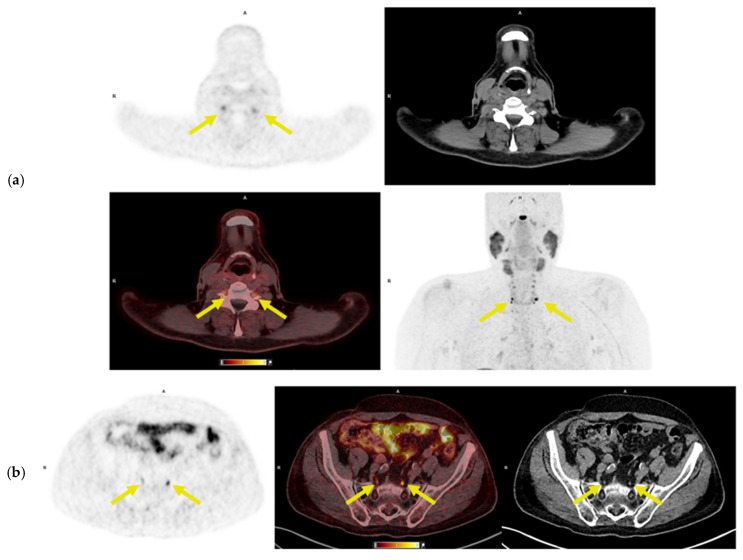
^64^Cu-DOTATATE PET, low-dose CT, fused images (PET/ldCT), and maximum intensity projection (MIP) of the cervical region (**a**) and pelvic region (**b**) from a patient with neuroborreliosis. Abbreviations: R = Right, H = Head, A = Anterior. (**a**): increased ^64^Cu-DOTATATE uptake in cervical and upper thoracic ganglia aligns with upper extremity symptoms of bilateral shoulder pain and finger paresthesia. PET/CT shows uptake (SUV_max_ = 4.6) in the cervical and upper thoracic dorsal root ganglia (yellow arrows). (**b**): increased ^64^Cu-DOTATATE uptake in the lumbosacral ganglia corresponds to lower extremity symptoms in the same patient as (**a**). PET/CT shows uptake (SUV_max_ = 5.6) in the lumbosacral ganglia (yellow arrows), matching clinical findings of left leg pain and paresis.

A distinct pattern of symmetric ^64^Cu-DOTATATE uptake was observed in the dorsal root and paravertebral ganglia at multiple cervical spine levels in 8 NB patients and 1 control (*p* = 0.3). As a post hoc analysis, SUV_max_ and SUV_mean_ of the ganglia were measured in all patients. The median SUV_max_ in NB patients was 2.5 (range 1.8–4.6), compared to SUV_max_ 1.6 in the control (Figure 2). The control had erythema migrans but normal CSF findings. In addition, lumbosacral ganglia uptake was observed in 9 of 15 patients, with a median SUV_max_ of 3.4 (range 2.4–5.6), whereas no such uptake was found in the controls (*p* = 0.04).

Symptom patterns generally corresponded to the anatomical distribution of ^64^Cu-DOTATATE uptake (Table 2). Among 15 NB patients, 10 showed uptake in cervical or lumbosacral ganglia, all with at least partial symptom correspondence. Additionally, 4 patients had uptake confined to the stellate ganglia, of whom 3 showed symptom correspondence.

### 3.3. MRI

Enhancement findings suggestive of NB were observed in 5 of 15 NB patients. Two exhibited enhancements of cranial nerves, while two had the enhancement of the lumbar nerve roots of the cauda equina. One patient showed contrast enhancement in the cranial nerves, cauda equina, and cervical nerve roots. Among the remaining 10 patients, 1 displayed two minuscule, punctate intramedullary enhancements, not thought to be attributed to NB, while the other 9 had no MRI findings suggestive of NB. No pathological contrast enhancements were observed in the control group.

### 3.4. Quality of Life

The comparison between NB patients and controls across various health and cognitive measures showed no significant differences (Supplementary Figure S1). Median values for SF-36 variables—emotional well-being, energy and fatigue, and general health—as well as most MF-20 fatigue variables were nearly identical, with considerable interquartile range overlap. Some variation was observed in pain, physical function, social function, and MOCA scores, but the differences were not statistically significant.

**Figure 3:**
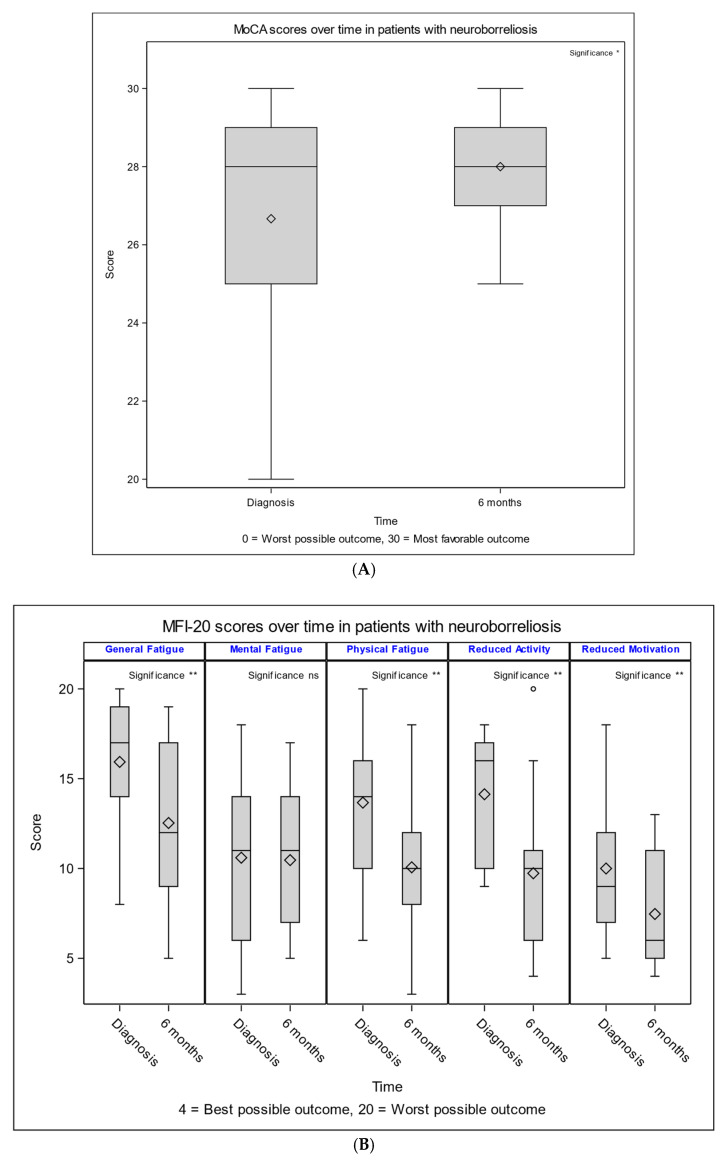

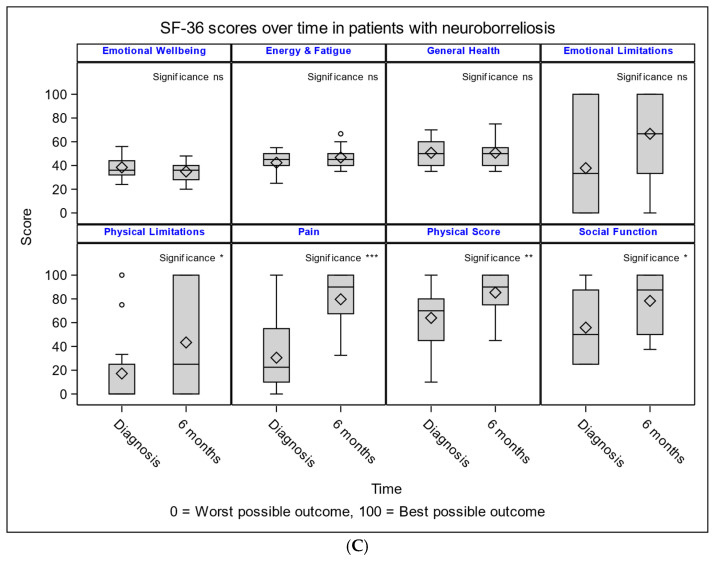
Boxplots showing changes in score diagnosis to a 6-month follow-up in patients with neuroborreliosis (**A**) Montreal Cognitive Assessment (MoCA) scores; (**B**) Multidimensional Fatigue Inventory-20 (MFI-20); and (**C**) Short Form 36 (SF-36). (**A**): MoCA scores assessing cognitive function with higher scores six months after diagnosis. (**B**): MFI-20 subscales with improving scores of general fatigue, physical fatigue, reduced activity, and reduced motivation six months after diagnosis. (**C**): SF-36 domains improving scores of physical score, physical limitations, pain, and social functioning six months after diagnosis. Boxplots show the interquartile range (IQR), with horizontal lines representing the median, diamonds representing the mean, and whiskers extending to 1.5× IQR. Circles indicate outliers. Significant changes over time are denoted by asterisks (* *p* < 0.05, ** *p* < 0.01, and *** *p* < 0.001, ns = not significant).

From diagnosis to a six-month follow-up, NB patients showed improvements in cognitive performance (MOCA +1 IQR −1 to 3), reductions in fatigue (MFI-20: general fatigue −2, IQR −7 to −1, physical fatigue −4, IQR −6 to −1, reduced activity −5, IQR −7 to 0, reduced motivation −3, and IQR −5 to −1), and improvements in physical functioning (12, IQR 0 to 35) and pain (55, IQR 12.5 to 80). Mental fatigue and other SF-36 domains showed minimal or no significant changes (Figure 3).

## 4. Discussion

This is the first study to explore ^64^Cu-DOTATATE PET/CT in NB. One patient showed very discrete focal uptake corresponding with MRI enhancement and facial palsy, limiting its diagnostic utility. However, a pattern of symmetric ^64^Cu-DOTATATE uptake was seen in the dorsal root and paravertebral ganglia at multiple cervical and lumbosacral levels in 10 of 15 NB patients. An additional four patients showed uptake confined to the stellate ganglia. In total, 13 patients had symptoms aligning with the uptake distribution. The clinical relevance of this ganglionic uptake remains uncertain. However, its alignment with symptom distribution and presence in only one control patient (with erythema migrans) suggests immune activation or altered ganglionic physiology.

Our findings did not support the hypothesis that ^64^Cu-DOTATATE PET would improve diagnostic accuracy in NB. Only one patient had focal ^64^Cu-DOTATATE uptake concordant with clinical symptoms and MRI findings, emphasizing the limited anatomical correlation between PET and MRI. This may be due to the small size and low uptake of inflammatory lesions, which limit detectability. Although the study did not reach the pre-specified statistical power, the minimal differences observed between groups suggest that any true effect is likely small or clinically insignificant.

Prior studies have shown that while MRI can occasionally indicate NB through cranial nerve, nerve root, or leptomeningeal enhancement, such findings are inconsistent and present in only 30–40% of cases. Cranial CT has no diagnostic value in NB [17,18] and PET/CT with ^18^Fluorine-fluorodeoxyglucose (^18^F-FDG) has shown inconsistent findings, ranging from cerebral hypometabolism, hypermetabolism, and focal brainstem inflammation to normal scans [20,21,22]. Although widely used for imaging inflammation, ^18^F-FDG lacks specificity, as it is taken up by many metabolically active cells, including immune and non-immune cells [23]. High physiological brain uptake further limits its utility in detecting meningeal inflammation. In contrast, ^64^Cu-DOTATATE targets SSTR2, predominantly expressed on activated macrophages, offering greater cellular specificity and lower CNS background. No head-to-head comparisons of FDG and DOTATATE in NB currently exist, but future studies are needed to evaluate their respective diagnostic roles and potential complementarity. More recently, PET imaging using the translocator protein (TSPO) tracer [^11^C]N,N-diethyl-2-(2-(4-(2-fluoroethoxy)phenyl)-5,7-dimethylpyrazolo [1,5-a]pyrimidin-3-yl)acetamide ([^11^C]DPA-713) has been used to assess glial activation and neuroinflammation. Increased tracer binding has been observed in patients with persistent symptoms following treatment [24].

The temporal delay between the initiation of antibiotic therapy and the ^64^Cu-DOTATATE PET/CT scan is important to consider. Effective antibiotic treatment likely reduces the inflammatory response, thereby lowering tracer uptake. Notably, CSF pleocytosis has been shown to decrease rapidly, by approximately 70–80%, within three weeks of antibiotic treatment [25]. All NB patients in our study received antibiotic treatment prior to PET/CT imaging, with a median duration of 10 days. In contrast, the animal study on Lyme arthritis involved no antibiotic intervention [16], and the endocarditis patients had received a mean of five days of antibiotic treatment at the time of imaging [11]. The antibiotic treatment likely weakened the inflammatory activity, leading to further reduction in tracer signal. Although this may limit the sensitivity of ^64^Cu-DOTATATE PET/CT in detecting active NB disease, postponing antibiotic treatment for imaging purposes would neither be ethically acceptable nor clinically relevant.

Macrophages play a central role in the immune response to *B. burgdorferi*, with infiltration shown in skin lesions from erythema migrans. The increased mononuclear cell counts in CSF and the local production of soluble CD163 (sCD163) support macrophage activation during NB [26,27,28]. Infected rhesus macaques also demonstrate macrophages in the meninges and brain, though in lower numbers than B- and T-lymphocytes, suggesting a limited role in early CNS involvement [29]. However, at later stages, macrophages become the predominant cell type in brain lesions [30]. Together with our findings, these studies suggest that in early NB, macrophage presence may be too limited—or lesions too small—to produce a detectable ^64^Cu-DOTATATE signal. In the single NB case with focal ^64^Cu-DOTATATE uptake, SUV_max_ was only 0.9. This is low compared to uptake in prosthetic valve endocarditis (IQR: 2.02–3.86) and markedly lower than in NETs, where mean SUV_max_ reaches 62.2 due to dense SSTR2 expression [10,11].

An unexpected observation was the symmetrical focal uptake in the dorsal root ganglia and paravertebral ganglia at multiple cervical and lumbosacral levels in most NB patients and one control. Animal studies have confirmed the physiological expression of SSTR2 in the dorsal root ganglia [31], and case reports have documented physiological uptake of ^68^Ga-DOTATATE—another SSTR2 targeting tracer—in the right stellate ganglion [32]. Advances in high-resolution, low-noise PET scanners have improved detection of such subtle signals. A recent review on total-body PET/CT identified DOTA-ligand uptake in paraspinal ganglia as a potential imaging pitfall, supporting the idea that some ganglionic uptake may be physiological [33]. The use of a LAFOV PET scanner provided high sensitivity and enabled whole-body imaging. Such systems may support lower-dose protocols and future kinetic or dynamic studies [34]. However, both infection and inflammation can increase ganglionic uptake by modulating SSTR expression or recruiting SSTR2-expressing immune cells [11,12,31].

As ^64^Cu-DOTATATE is a peptide, its ability to cross the intact blood–brain barrier is limited, similar to conventional MRI contrast agents [35]. Consequently, both tracer binding to brain microglia and infiltration of systemically labeled macrophages to the brain depend on blood–brain barrier disruption. In contrast, the dorsal root ganglia are protected by the more permeable blood–dorsal root ganglia barrier, allowing easier tracer access [36]. This may explain the observed ganglionic uptake. Since the ganglionic uptake lacked MRI correlates, the diagnostic certainty is limited. Low or borderline SUV values could reflect normal variation or noise. Without anatomical confirmation, such uptake remains difficult to interpret. Future studies using PET/MRI or histopathologic validation could help confirm these subtle findings. The measured SUV_max_ values in our cohort were generally low and overlapped partially with those of controls. Neither the degree nor the pattern of uptake alone is sufficient to differentiate infection from normal physiology or generalized inflammation.

The higher frequency of symmetric ganglionic uptake in NB patients suggests a disease-related mechanism. The only control patient with cervical uptake had erythema migrans and systemic symptoms but normal CSF, raising the possibility that *B. burgdorferi*, like ^64^Cu-DOTATATE, more easily infiltrates the dorsal root ganglia than it crosses the blood–brain barrier. Such ganglionic involvement could represent an early stage of infection, preceding detectable CNS involvement. Supporting this, previous studies have described nerve-related symptoms before CSF pleocytosis, suggesting that peripheral nerve injury may precede measurable meningeal inflammation [2]. Similarly, a study on facial palsy in NB found that fewer than half of patients had CSF pleocytosis, and none showed proximal nerve involvement, indicating that nerve damage likely originates peripherally rather than from CNS inflammation [37].

The pathogenesis of NB remains incompletely understood and involves complex host–pathogen interactions beginning with tick transmission. *B. burgdorferi* escapes the circulating systemic immune response by disseminating to the CNS and triggers a localized immune response, evident by CSF pleocytosis [28,38]. Hematogenous spread across the blood–CSF barrier is the most widely supported route of CNS entry [2]. However, the association between neurological symptoms and tick bites also raises the possibility of peripheral nerve dissemination [39]. The neurotropic behavior of *B. burgdorferi* and ability to evade immune surveillance suggest that peripheral ganglia could serve as intermediate sites of colonization, with potential spillover into the CNS. This is similar to herpesviruses, which can persist in autonomic ganglia and later reactivate with CNS involvement [40]. The most common symptoms of NB are radiating pains accompanied by sensory deficits, weakness or even palsy [3,5]. Although NB typically presents with mononuclear pleocytosis, the extent of CNS infiltration and infection remains unclear due to limited access to CNS tissues and the biological heterogeneity of *B. burgdorferi* [28,41]. Consistent with common symptoms and suggestive of dorsal root ganglia involvement, animal studies have demonstrated the presence of spirochetes and inflammation within the dorsal root ganglia with neurodegeneration and significant neuronal and satellite glial cell apoptosis [41,42]. The observed ganglia uptake on ^64^Cu-DOTATATE PET imaging appears clinically relevant, since the uptake corresponded to symptom distribution, such as cervical uptake in patients with upper extremity pain and lumbar uptake in those with lower extremity symptoms, supporting a link between tracer accumulation and localized immune activation.

Although some variation was observed in pain, physical functioning, social functioning, and MoCA scores, overall comparisons between NB patients and controls showed no statistically significant differences across general health, fatigue, and cognitive measures. Symptoms such as sensory disturbances, dizziness, headaches, and musculoskeletal pain were reported with similar frequency in both groups; however, NB patients more often reported radiating pain, cranial nerve palsies, and limb paresis. Over six months, NB patients showed improvements in cognitive performance, fatigue, physical function, and social functioning, while changes in mental fatigue and emotional domains were minimal. These results highlight the generally favorable prognosis of NB and are consistent with previous studies that report persistent mental and emotional symptoms in a subset of well-treated patients [43,44].

### Strength and Limitations

One strength of this study is the well-defined patient cohort, which provides a clear clinical framework for evaluating ^64^Cu-DOTATATE PET/CT and MRI in suspected NB. The study offers valuable exploratory insights into the pathogenesis of *B. burgdorferi.* However, the interim analysis only found one patient with relevant, localized ^64^Cu-DOTATATE uptake, and the study was terminated after the inclusion of 15 NB patients. Antibiotic treatment before imaging may have influenced the results, and a shorter treatment duration could have provided a more accurate assessment of disease activity. Additionally, the heterogeneity of the control group represents a limitation that may affect interpretation of tracer uptake and comparisons between groups.

## 5. Conclusions

This study is the first to evaluate the use of ^64^Cu-DOTATATE PET/CT in patients with suspected NB. An unexpected finding was the symmetric uptake observed in the dorsal root and paravertebral ganglia in 10 of 15 patients. While the clinical relevance of this uptake remains uncertain, it may reflect early peripheral immune activation or involvement in the dissemination of *B. burgdorferi* to the CNS. Only one patient demonstrated very faint focal uptake corresponding with MRI enhancement and clinical signs; overall, ^64^Cu-DOTATATE PET/CT did not contribute additional diagnostic value beyond standard clinical evaluation.

## Figures and Tables

**Table 1:** Baseline characteristics of 15 NB patients and 5 controls prior to imaging.

Characteristics	All Participants (*n* = 20)	NB Patients ^1^ (*n* = 15)	Controls ^2^ (*n* = 5)
Age, years, median (IQR)	56 (48–66)	58 (51–65)	53 (39–66)
Sex, female (%)	7 (35)	5 (33)	2 (40)
BMI, median (IQR)	23.9 (22.9–27.6)	23.5 (22.0–26.0)	28.7 (24.7–29.1)
Charlson Comorbidity Index, median (IQR)	2 (1–3)	2 (1–3)	1 (0–2)
History of tick bite (yes, %)	14 (70)	10 (67)	4 (80)
Symptom duration before lumbar puncture, *n* (%)			
0–14 days	3 (15)	1 (7)	2 (40)
15–44 days	13 (65)	11 (73)	2 (40)
45–179 days	4 (20)	3 (20)	1 (20)
Days of antibiotic treatment before PET/CT, median (IQR) ^3^	9.5 (7–11)	10 (6–11)	8 (7–16)
Self-reported symptoms (yes, %)			
Radiating pain	10 (50)	9 (60)	1 (20)
Facial/eye nerve paralysis	5 (25)	5 (33)	0 (0)
Limb paresis	3 (15)	3 (20)	0 (0)
Facial pain/sensory changes	3 (15)	2 (13)	1 (20)
Sensory disturbances	10 (50)	7 (47)	3 (60)
Headache	9 (45)	7 (47)	2 (40)
Fatigue	17 (85)	13 (87)	4 (80)
Dizziness	11 (55)	8 (53)	3 (60)
Muscle pain	12 (60)	10 (67)	2 (40)
Joint pain	8 (40)	6 (40)	2 (40)
Memory/concentration issues	8 (40)	7 (46)	1 (20)
Altered bowel movement	4 (20)	3 (20)	1 (20)
Changes in hearing or taste	5 (25)	4 (27)	1 (20)
CSF analysis			
Leucocytes, cells/µL, median (IQR)	110 (14–185)	155 (103–246)	1 (1–4)
Mononuclear cells/µL, median (IQR)	105 (15–188)	155 (102–246)	1 (1–5.5)
Polynuclear cells, cells/µL, median (IQR)	2 (1–4)	3 (2–5)	1 (0.5–1)
CXCL13 > 500 pg/mL (yes, %)	6 (30)	6 (40)	0 (0)
*B. burgdorferi* AI index (positive, %)	10 (50)	10 (67)	0 (0)
Blood analysis			
*B. burgdorferi* IgG (positive, %) missing	11 (69) (4)	9 (60) (4)	2 (40)
Elevated infectious parameters (yes, %) missing	5 (29) 2	4 (29) 1	1 (25) 1
Leucocytes, ×10^9^/L, median (IQR)	7.1 (5.9–8.5)	6.9 (5.8–8.5)	7.2 (5.9–8.4)
C-reactive protein, mg/L, median (IQR)	1.0 (1–5.6)	2.0 (1–5.6)	1 (1–4)

Abbreviations: CSF, cerebrospinal fluid; IQR, interquartile range; and NB, neuroborreliosis. Categorical variables are presented as *n* (%) and continuous variables as medians with interquartile rates (IQRs). ^1^ Among 15 NB patients, 5 were classified as possible NB (symptoms and pleocytosis) and 10 as definite NB (symptoms, pleocytosis, and intrathecal *Borrelia burgdorferi* antibody production); ^2^ Among the 5 controls, 1 was diagnosed with polymyalgia rheumatica, 1 with erythema migrans, and the remaining were completed without a diagnosis; ^3^ Among treated participants, two controls did not receive any antibiotic treatment.

**Table 2:** Distributions of ^64^Cu-DOTATATE uptake and symptoms.

^64^Cu-DOTATATE Uptake	Number of Patients	Main Symptoms	Number of Patients
Cervical ganglia	1	Headache	1
Stellate ganglia only	4	Ipsilateral facial palsy and ipsilateral radiating upper extremity pain	1
		Meningeal symptoms	1
		Ipsilateral facial palsy and contralateral radiating upper extremity pain	1
		Bilateral radiating lower extremity pain	1
Lumbosacral ganglia and stellate ganglia	2	Ipsilateral facial palsy, headache, and neck pain	1
		Ipsilateral lower extremity pain	1
Both cervical and lumbosacral	7	Upper extremity pain and paresis	1
		Ipsilateral facial palsy and upper extremity pain and paresis	1
		Ipsilateral facial palsy and upper extremity paresis	1
		Ipsilateral lower extremity pain and bilateral upper extremity sensory disturbances	1
		Thoracic and bilateral radiating lower extremity pain	1
		Ipsilateral facial palsy, thoracic, and bilateral radiating lower extremity pain	1
		Ipsilateral facial palsy and radiating lower extremity pain	1
No enhancement	1	Ipsilateral facial palsy	1
	Number of controls	Main symptoms	Number of controls
Cervical ganglia	1	Erythema migrans and ipsilateral radiating neck pain	1
Stellate ganglia only	1	Bilateral facial sensory disturbances	1
Lumbosacral ganglia and stellate ganglia	0		NA
Both cervical and lumbosacral	0		NA
No enhancement	3	Headache and neck pain	2
		Neck pain; sensory disturbances	1

## Data Availability

The data that support the findings of this study are available from the corresponding author, M.Ø., upon reasonable request.

## Supplementary Materials

The following supporting information can be downloaded at https://www.mdpi.com/article/10.3390/diagnostics16040561/s1, Figure S1: comparison of baseline scores between patients with neuroborreliosis (NB) and healthy controls.

## Abbreviations

The following abbreviations are used in this manuscript:

*B. burgdorferi*	*Borrelia burgdorferi*
CNS	Central nervous system
CSF	Cerebrospinal fluid
CT	Computed tomography
EFNSs	European Federation of Neurological Societies
IQR	Interquartile range
LAFOV	Long axial field of view
MBq	Megabecquerel
MFI-20	Multidimensional Fatigue Inventory-20
MIP	Maximum intensity projection
MoCA	Montreal Cognitive Assessment
MRI	Magnetic resonance imaging
NB	Neuroborreliosis
NET	Neuroendocrine tumor
PET	Positron emission tomography
PSF	Point spread function
REDCap	Research Electronic Data Capture
SF-36	Short Form (36) Health Survey
SSTR2	Somatostatin receptor subtype 2
SUV_max_	Maximum standardized uptake value
SUV_mean_	Mean standardized uptake value

## References

[1] Mygland A., Ljostad U., Fingerle V., Rupprecht T., Schmutzhard E., Steiner I. (2010). EFNS guidelines on the diagnosis and management of European Lyme neuroborreliosis. Eur. J. Neurol..

[2] Halperin J.J., Eikeland R., Branda J.A., Dersch R. (2022). Lyme neuroborreliosis: Known knowns, known unknowns. Brain.

[3] Hansen K., Lebech A.M. (1992). The clinical and epidemiological profile of Lyme neuroborreliosis in Denmark 1985–1990. A prospective study of 187 patients with Borrelia burgdorferi specific intrathecal antibody production. Brain.

[4] Solheim A.M., Skarstein I., Quarsten H., Lorentzen Å.R., Berg-Hansen P., Eikeland R., Reiso H., Mygland Å., Ljøstad U. (2024). Clinical and laboratory characteristics during a 1-year follow-up in European Lyme neuroborreliosis: A prospective cohort study. Eur. J. Neurol..

[5] Knudtzen F.C., Andersen N.S., Jensen T.G., Skarphédinsson S. (2017). Characteristics and Clinical Outcome of Lyme Neuroborreliosis in a High Endemic Area, 1995–2014: A Retrospective Cohort Study in Denmark. Clin. Infect. Dis..

[6] Stupica D., Bajrović F.F., Blagus R., Cerar Kišek T., Collinet-Adler S., Lah A., Levstek E., Ružić-Sabljić E. (2021). Clinical manifestations and long-term outcome of early Lyme neuroborreliosis according to the European Federation of Neurological Societies diagnostic criteria (definite versus possible) in central Europe. A retrospective cohort study. Eur. J. Neurol..

[7] Nordberg C.L., Bodilsen J., Knudtzen F.C., Storgaard M., Brandt C., Wiese L., Hansen B.R., Andersen Å.B., Nielsen H., Lebech A.M. (2020). Lyme neuroborreliosis in adults: A nationwide prospective cohort study. Ticks Tick. Borne Dis..

[8] Krüger H., Reuss K., Pulz M., Rohrbach E., Pflughaupt K.W., Martin R., Mertens H.G. (1989). Meningoradiculitis and encephalomyelitis due to Borrelia burgdorferi: A follow-up study of 72 patients over 27 years. J. Neurol..

[9] Vrijmoeth H.D., Ursinus J., Harms M.G., Tulen A.D., Baarsma M.E., Van De Schoor F.R., Gauw S.A., Zomer T.P., Vermeeren Y.M., Ferreira J.A. (2023). Determinants of persistent symptoms after treatment for Lyme borreliosis: A prospective observational cohort study. eBioMedicine.

[10] Carlsen E.A., Johnbeck C.B., Binderup T., Loft M., Pfeifer A., Mortensen J., Oturai P., Loft A., Berthelsen A.K., Langer S.W. (2020). ^64^Cu-DOTATATE PET/CT and Prediction of Overall and Progression-Free Survival in Patients with Neuroendocrine Neoplasms. J. Nucl. Med..

[11] Hadji-Turdeghal K., Fosbøl M., Hasbak P., Löfgren J., Bull Rasmussen I., Bundgaard H., Iversen K., Bruun N.E., Møller C.H., Tuxen C. (2025). First-In-Human Study of [(64)Cu]Cu-DOTATATE PET/CT in Infective Endocarditis: A Prospective Head-to-Head Comparison With [(18)F]FDG. Circ. Cardiovasc. Imaging.

[12] Jensen J.K., Madsen J.S., Jensen M.E.K., Kjaer A., Ripa R.S. (2023). [^64^Cu]Cu-DOTATATE PET metrics in the investigation of atherosclerotic inflammation in humans. J. Nucl. Cardiol..

[13] Nobashi T., Nakamoto Y., Kubo T., Ishimori T., Handa T., Tanizawa K., Sano K., Mishima M., Togashi K. (2016). The utility of PET/CT with 68Ga-DOTATOC in sarcoidosis: Comparison with 67Ga-scintigraphy. Ann. Nucl. Med..

[14] Verhaegh D., Joosten L.A.B., Oosting M. (2017). The role of host immune cells and Borrelia burgdorferi antigens in the etiology of Lyme disease. Eur. Cytokine Netw..

[15] Woitzik P., Linder S. (2021). Molecular Mechanisms of Borrelia burgdorferi Phagocytosis and Intracellular Processing by Human Macrophages. Biology.

[16] Skovsbo Clausen A., Ørbæk M., Renee Pedersen R., Oestrup Jensen P., Lebech A.M., Kjaer A. (2020). ^64^Cu-DOTATATE Positron Emission Tomography (PET) of Borrelia Burgdorferi Infection: In Vivo Imaging of Macrophages in Experimental Model of Lyme Arthritis. Diagnostics.

[17] Ørbæk M., Bodilsen J., Gynthersen R.M.M., Shekhrajka N., Nordberg C.L., Larsen L., Storgaard M., Brandt C., Wiese L., Hansen B.R. (2020). CT and MR neuroimaging findings in patients with Lyme neuroborreliosis: A national prospective cohort study. J. Neurol. Sci..

[18] Lindland E.S., Solheim A.M., Andreassen S., Quist-Paulsen E., Eikeland R., Ljostad U., Mygland A., Elsais A., Nygaard G.O., Lorentzen A.R. (2018). Imaging in Lyme neuroborreliosis. Insights Imaging.

[19] Loft M., Carlsen E.A., Johnbeck C.B., Johannesen H.H., Binderup T., Pfeifer A., Mortensen J., Oturai P., Loft A., Berthelsen A.K. (2021). ^64^Cu-DOTATATE PET in Patients with Neuroendocrine Neoplasms: Prospective, Head-to-Head Comparison of Imaging at 1 Hour and 3 Hours After Injection. J. Nucl. Med..

[20] Newberg A., Hassan A., Alavi A. (2002). Cerebral metabolic changes associated with Lyme disease. Nucl. Med. Commun..

[21] Plotkin M., Hautzel H., Krause B.J., Mohr S., Langen K.J., Muller H.W. (2005). Fluorine-18-labeled fluorodeoxyglucose-positron emission tomography studies of acute brainstem Lyme neuroborreliosis. J. Neurosurg..

[22] Ørbæk M., Klausen C., Lebech A.M., Mens H. (2020). Lyme Neuroborreliosis in a Patient with Breast Cancer: MRI and PET/CT Findings. Diagnostics.

[23] Glaudemans A.W., Slart R.H., van Dijl J.M., van Oosten M., van Dam G.M. (2015). Molecular imaging of infectious and inflammatory diseases: A terra incognita. J. Nucl. Med..

[24] Coughlin J.M., Yang T., Rebman A.W., Bechtold K.T., Du Y., Mathews W.B., Lesniak W.G., Mihm E.A., Frey S.M., Marshall E.S. (2018). Imaging glial activation in patients with post-treatment Lyme disease symptoms: A pilot study using [^11^C]DPA-713 PET. J. Neuroinflammation.

[25] Kortela E., Kanerva M.J., Puustinen J., Hurme S., Airas L., Lauhio A., Hohenthal U., Jalava-Karvinen P., Nieminen T., Finnilä T. (2021). Oral Doxycycline Compared to Intravenous Ceftriaxone in the Treatment of Lyme Neuroborreliosis: A Multicenter, Equivalence, Randomized, Open-label Trial. Clin. Infect. Dis..

[26] Ørbæk M., Gynthersen R.M.M., Mens H., Brandt C., Stenør C., Wiese L., Andersen Å.B., Møller H.J., Lebech A.M. (2023). Cerebrospinal fluid levels of the macrophage-specific biomarker sCD163 are diagnostic for Lyme neuroborreliosis: An observational cohort study. Clin. Chim. Acta.

[27] Brem C.E., Goldberg L.J. (2022). Early Erythema Migrans: Do Not Count on Plasma Cells. Am. J. Dermatopathol..

[28] Snik M.E., Stouthamer N., Hovius J.W., van Gool M.M.J. (2024). Bridging the gap: Insights in the immunopathology of Lyme borreliosis. Eur. J. Immunol..

[29] Ramesh G., Borda J.T., Gill A., Ribka E.P., Morici L.A., Mottram P., Martin D.S., Jacobs M.B., Didier P.J., Philipp M.T. (2009). Possible role of glial cells in the onset and progression of Lyme neuroborreliosis. J. Neuroinflammation.

[30] Roberts E.D., Bohm R.P., Lowrie R.C., Habicht G., Katona L., Piesman J., Philipp M.T. (1998). Pathogenesis of Lyme neuroborreliosis in the rhesus monkey: The early disseminated and chronic phases of disease in the peripheral nervous system. J. Infect. Dis..

[31] Bär K.J., Schurigt U., Scholze A., Segond Von Banchet G., Stopfel N., Bräuer R., Halbhuber K.J., Schaible H.G. (2004). The expression and localization of somatostatin receptors in dorsal root ganglion neurons of normal and monoarthritic rats. Neuroscience.

[32] Berg Z., Koppula B.R. (2019). ^68^Ga-DOTATATE Uptake by Cervicothoracic (Stellate) Ganglia. Clin. Nucl. Med..

[33] Mingels C., Chung K.J., Pantel A.R., Rominger A., Alberts I., Spencer B.A., Nardo L., Pyka T. (2025). Total-Body PET/CT: Challenges and Opportunities. Semin. Nucl. Med..

[34] Urso L., Frantellizzi V., Vincentis G.D., Schillaci O., Filippi L., Evangelista L. (2023). Clinical applications of long axial field-of-view PET/CT scanners in oncology. Clin. Transl. Imaging.

[35] Heye A.K., Culling R.D., Valdés Hernández Mdel C., Thrippleton M.J., Wardlaw J.M. (2014). Assessment of blood-brain barrier disruption using dynamic contrast-enhanced MRI. A systematic review. Neuroimage Clin..

[36] Jimenez-Andrade J.M., Herrera M.B., Ghilardi J.R., Vardanyan M., Melemedjian O.K., Mantyh P.W. (2008). Vascularization of the dorsal root ganglia and peripheral nerve of the mouse: Implications for chemical-induced peripheral sensory neuropathies. Mol. Pain.

[37] Halperin J.J. (2003). Facial nerve palsy associated with lyme disease. Muscle Nerve Off. J. Am. Assoc. Electrodiagn. Med..

[38] Aslam B., Nisar M.A., Khurshid M., Farooq Salamat M.K. (2017). Immune escape strategies of Borrelia burgdorferi. Future Microbiol..

[39] Ogrinc K., Kastrin A., Lotrič-Furlan S., Bogovič P., Rojko T., Maraspin V., Ružić-Sabljić E., Strle K., Strle F. (2022). Colocalization of Radicular Pain and Erythema Migrans in Patients With Bannwarth Syndrome Suggests a Direct Spread of Borrelia Into the Central Nervous System. Clin. Infect. Dis..

[40] Hakami M.A., Khan F.R., Abdulaziz O., Alshaghdali K., Hazazi A., Aleissi A.F., Abalkhail A., Alotaibi B.S., Alhazmi A.Y.M., Kukreti N. (2024). Varicella-zoster virus-related neurological complications: From infection to immunomodulatory therapies. Rev. Med. Virol..

[41] Ramesh G., Didier P.J., England J.D., Santana-Gould L., Doyle-Meyers L.A., Martin D.S., Jacobs M.B., Philipp M.T. (2015). Inflammation in the pathogenesis of lyme neuroborreliosis. Am. J. Pathol..

[42] Cadavid D., O’Neill T., Schaefer H., Pachner A.R. (2000). Localization of Borrelia burgdorferi in the nervous system and other organs in a nonhuman primate model of lyme disease. Lab. Investig..

[43] Foret J., Paren A.J., Zayet S., Chirouze C., Gendrin V., Bouiller K., Klopfenstein T. (2025). Residual Symptoms and Quality of Life After Treated Lyme Neuroborreliosis: Case-Control Study (QoLYME). Open Forum Infect. Dis..

[44] Andreassen S., Lindland E.M.S., Beyer M.K., Solheim A.M., Ljøstad U., Mygland Å., Lorentzen Å.R., Reiso H., Bjuland K.J., Pripp A.H. (2023). Assessment of cognitive function, structural brain changes and fatigue 6 months after treatment of neuroborreliosis. J. Neurol..

