# Supplementary material for: 64Cu-DOTATATE-PET/CT in Neuroborreliosis Shows Increased Tracer Uptake in Dorsal Root and Paravertebral Ganglia
Source: Diagnostics (Basel). 2026 Feb 13;16(4):561. doi: 10.3390/diagnostics16040561 (PMC12939385; doi:10.3390/diagnostics16040561)
Supplement: Supplementary file 1 [file diagnostics-16-00561-s001.zip › diagnostics-4076882-supplementary.pdf]

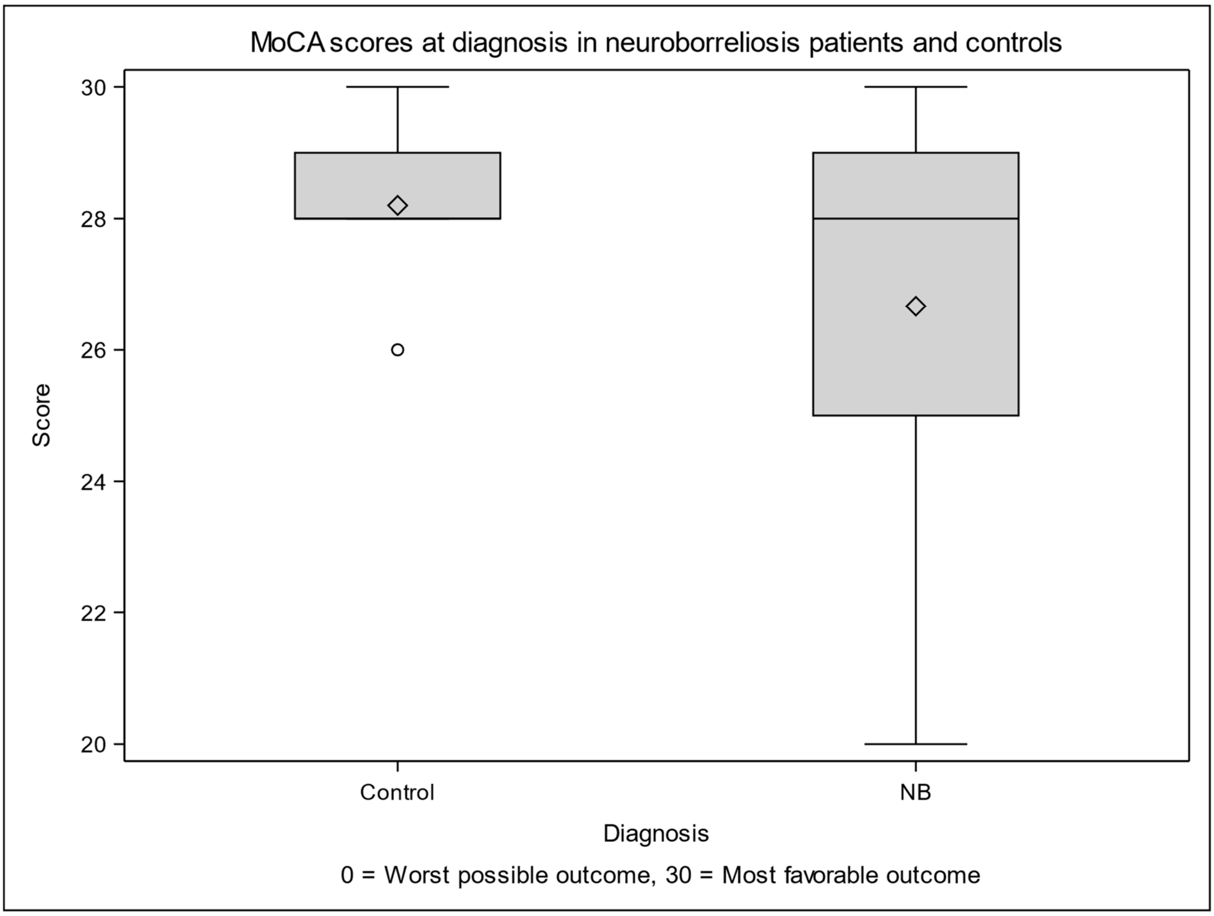
MoCA scores at diagnosis in neuroborreliosis patients and controls
30
28
26
24
22
20
Score
Control
NB
Diagnosis
0 = Worst possible outcome, 30 = Most favorable outcome

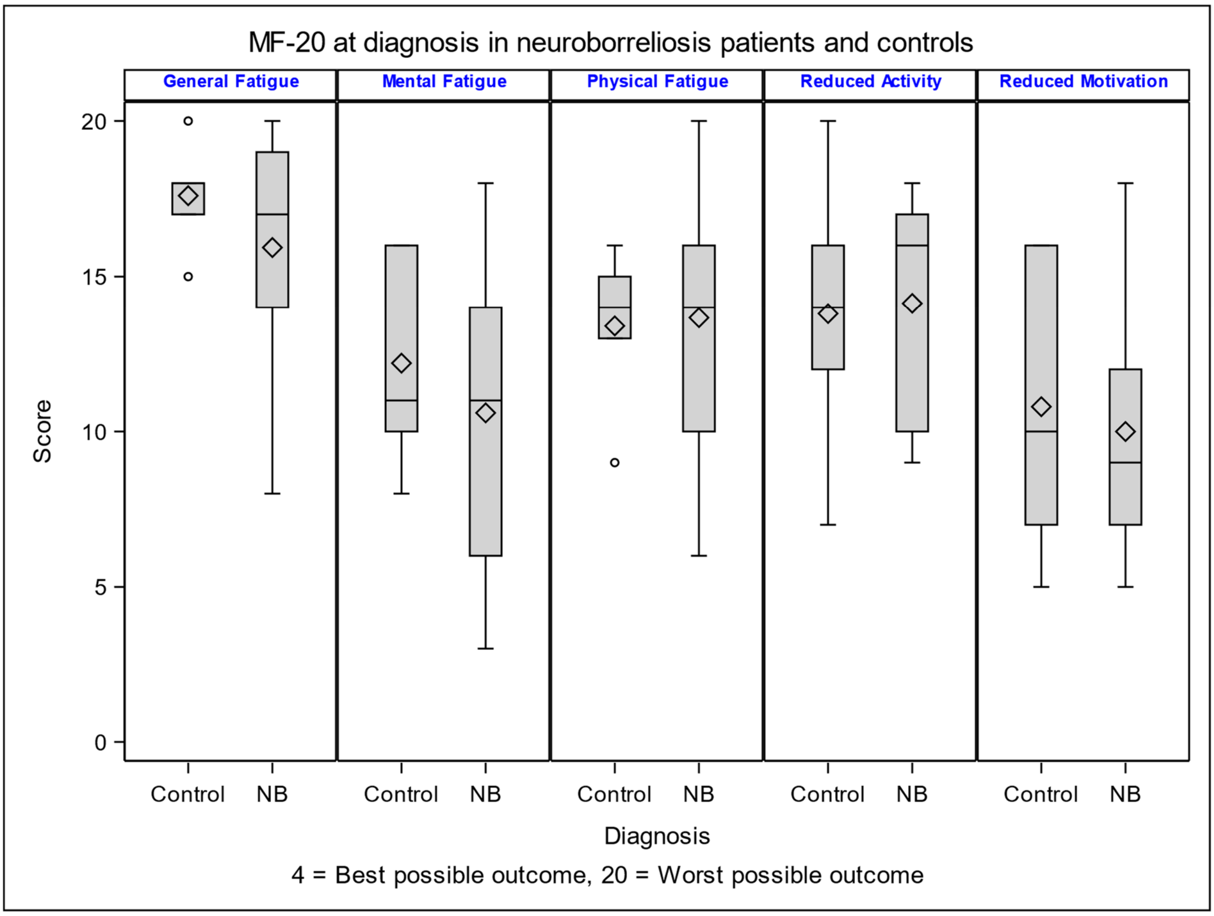
MF-20 at diagnosis in neuroborreliosis patients and controls
General Fatigue
Mental Fatigue
Physical Fatigue
Reduced Activity
Reduced Motivation
Score
20
15
10
5
0
Control
NB
Diagnosis
4 = Best possible outcome, 20 = Worst possible outcome

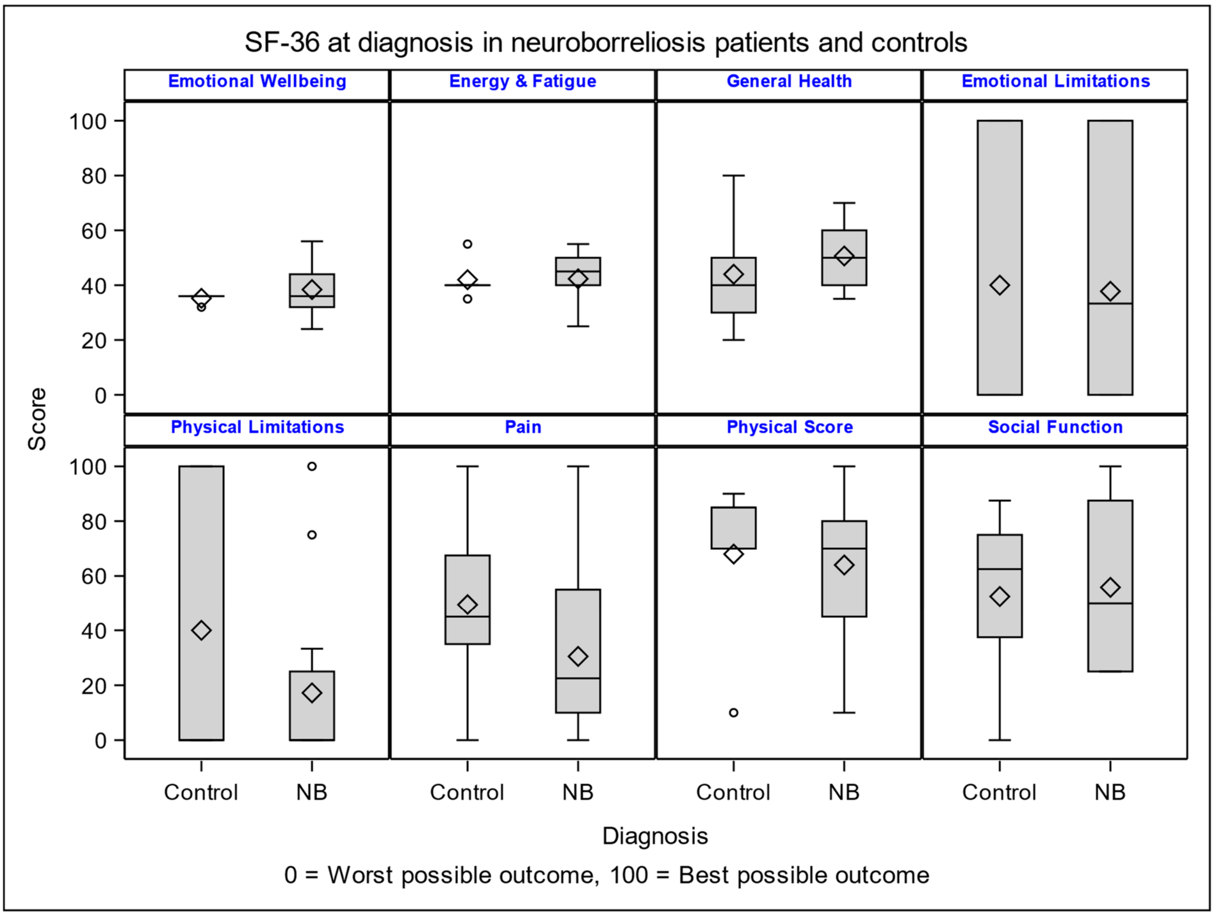

Supplemental Fig. S1. Boxplots comparing baseline scores between patients with neuroborreliosis (NB) and healthy controls. Panel A: Montreal Cognitive Assessment (MoCA) scores (cognitive function). Panel B: Multidimensional Fatigue Inventory (MFI-20) subscales (general fatigue, physical fatigue, mental fatigue, reduced activity, and reduced motivation). Panel C: Short Form-36 (SF-36) domains (emotional wellbeing, energy/fatigue, physical role limitation, physical functioning, pain, social functioning, and general health). Boxes represent the interquartile range (IQR), horizontal lines indicate the median, and whiskers represent 1.5× IQR.
